# Zhongdanyaozhi No. 1 and Zhongdanyaozhi No. 2 Are Hybrid Cultivars of *Salvia miltiorrhiza* with High Yield and Active Compounds Content

**DOI:** 10.1371/journal.pone.0162691

**Published:** 2016-09-22

**Authors:** Meng Chen, Chengmin Yang, Chun Sui, Yue Jin, Jianhe Wei

**Affiliations:** Institute of Medicinal Plant Development, Chinese Academy of Medical Sciences & Peking Union Medical College, Beijing, China; Suzhou University, CHINA

## Abstract

*Salvia miltiorrhiza* Bunge is an important medicinal plant used for the treatment of cardiovascular disease. Intraspecific hybridization between a male sterile line and inbred lines was followed by 39 F1 crossings. Cultivars “Zhongdanyaozhi No. 1” (ZD1) and “Zhongdanyaozhi No. 2” (ZD2) were obtained. In 2012 and 2013 tests in Beijing, the two cultivars were compared with three widely accepted types, SDCK, SXCK and HNCK from Shandong, Shanxi and Henan provinces. The yield of ZD1 and ZD2 exceeded the three CKs by more than 48.2% and 39.2%, respectively; the composition of the two hybrid cultivars was similar to the control, although the content of some compounds varied to some extent. The content of salvianolic acid B and tanshinone II A of both ZD1 and ZD2 could measure up the requirement of *Chinese Pharmacopoeia*. The former showed no obvious advantage than the three CKs, while the later’s tanshinone II A was 29.6% higher than the three CKs. Taken together, ZD1 is a high yielding and thick-root-type cultivar which is suitable for decoction pieces; while ZD2 is suitable for component especially lipophilic component extraction. ZD1 and ZD2 reported here are the first cultivars obtained by the hybridization of *S*. *miltiorrhiza*.

## Introduction

*Danshen*, the dried root of *Salvia miltiorrhiza* Bunge, is highly valued in Traditional Chinese Medicine either used alone or in combination with other herbs. It has been widely used for the treatment of various diseases, including coronary heart disease, cerebrovascular disease, Alzheimer’s disease, Parkinson’s disease, renal deficiency, hepatocirrhosis, cancer, and bone loss [1–3]. In Japan, the United States, and some European countries, *Danshen* is also employed as a new dietary supplement and is available in herbal shops [4]. Hydrophilic phenolic acids and lipophilic diterpenoid constituents have been determined to be the major constituents of *Danshen* [5], both of which actively function in cardio protection [6]. Hydrophilic compounds (including danshensu, rosmarinic acid, salvianolic acid B) play an effective role in scavenging peroxides and inhibiting the expression of adhesion molecules in vascular endothelium and leucocytes [7], while lipophilic compounds (including dihydrotanshinone I, tanshinone I, cryptotanshinone and tanshinone II A) are involved in antimicrobial activity [8] and endocrine regulation [9]. According to *The 2010 edition of Chinese Pharmacopoeia*, the content of salvianolic acid B and tanshinone II A of *Danshen* should be no less than 3.0% and 0.2%, respectively [10].

Currently, *S*. *miltiorrhiza* is commonly cultivated for medicinal and health purposes. Because of the loss of wild resources, China has facilitated the cultivation of *S*. *miltiorrhiza* in Shandong, Henan, Sichuan and Shaanxi provinces since the 1970s [11]; meanwhile, *S*. *miltiorrhiza* has also been introduced into other countries and districts, such as Germany [12], Australia [13, 14], and South Korea [15]. Although eleven cultivars have been reported up to now, none of them has found a broad plantation [16]. The dry root yield/667 m^2^ of *S*. *miltiorrhiza* in Henan and Shanxi ranged between 200 and 372 kg, while that in Shandong was 250–465 kg [17]. The performance of some strains was unsteady in the multi-region test, leading to the significate content variation of active compounds, especially lipophilic compounds, and some strains even cannot measure up according to *The Chinese Pharmacopoeia* [18]. In addition, some cultivars for specific usage like component extraction and decoction pieces are absent in production.

Heterosis breeding for medicinal plant species has been reported, including *Artemisia annu* [19], *Papaver somniferum* [20, 21], *Carthamus tinctorius* [22], *Platycodon grandiflorum* [23, 24], *Origanum majorana* [25], and *Petasites hybridus* [26]. Here, we report on the breeding of two hybrid cultivars of *S*. *miltiorrhiza*, “Zhongdanyaozhi No. 1” (ZD1) and “Zhongdanyaozhi No. 2” (ZD2), using the self-bred lines and a male sterile line. ZD1 and ZD2, which have been officially authenticated in China, show strong heterosis on the root yield and quality trait. They have proved the first successful hybridization of *S*. *miltiorrhiza*.

## Materials and Methods

In 2007, *S*. *miltiorrhiza* plants were collected from Shandong, Henan and Shanxi, the three major *S*. *miltiorrhiza*-producing provinces of China, and subsequently planted in Beijing, from which six male-sterile plants were found in 2009 and then crossed to different plants to determine the heredity of the fertility. The selected maintainers were self-bred for three generations, where the male sterile line was steadily produced. In 2012, three male-sterile lines were assessed and selected based on the flowering period, plant vigor and rate of overwintering survival. During this period of time, 17 self-bred lines were assessed and selected for the root yield, active compounds content and pollen quantity by the three-generation purification. In May to June of 2012, cross-fertilization was configured by 17 self-bred strains and 3 male-sterile strains into a total of 39 cross combinations which were used for the comparative test during 2012–2013. The seeds were harvested 3 weeks after pollination. In August 2012, the seeds of the 39 cross combinations were sowed in the field at the Institute of Medicinal Plant Development (IMPLAD), Beijing (N 40°01′, E 116°16′) and transplanted in April 2013. Then a randomized complete block design with 3 replicates was used, where the area of each repetition was 18 m × 4 m, and the plant density was 25 cm × 25 cm (row spacing × plant spacing), under daily field management as conducted in the major *S*. *miltiorrhiza-*producing areas [27]. Among these combinations, two showed outstanding root yield and active compounds content, DF807-5-1-5(♂) × DPS101 (♀) and DF804-17-1-1(♂) × DPS101 (♀), which were finally used to produce the hybrids named ZD1 and ZD2. In 2013–2014, ZD1 and ZD2 were further compared for their performance with the parent lines and three widely accepted cultivation types, SDCK, SXCK and HNCK, from Shandong, Shanxi and Henan provinces in China; additionally, they were planted in Henan (*Danshen* GAP plantation base of *Baiyunshan*) (N 34°30′, E 101°32′) and Shanxi (*Danshen* GAP plantation base of *Tasly*) (N 35°51′, E 109°29′) to evaluate their adaptability. The root samples were dried in an oven to constant weight at 60°C for further measurement.

### Plant characteristics

All plant characteristics, except the root skin color and average roots branch number, were measured in mid-June 2013, whereas the root skin color and average roots branch number were evaluated in mid-Nov 2013. Ten plants with three replicates per cultivar were measured and Tukey-Kramer tests were conducted to compare the means and variances.

### Yield evaluation

The dry root weight of 10 single plants was measured, and the yield of 667 m^2^ (Y/667 m^2^) was calculated based on the planting density. Decoction-pieces grade root ratio (DPR) was calculated as follows: DPR = decoction-pieces grade root number / total root number*100%, where decoction-pieces grade root number is the number of dry roots of more than 0.5 cm in diameter, and total root number is the number of dry roots of no less than 0.2 cm in diameter. Tukey-Kramer tests were conducted to compare the means and variances.

### Measurement of active compounds content

The contents of danshensu *(Dss*), rosmarinic acid (*Ra*), salvianolic acid B (*Sal B*), tanshinone I (*Ts I*), cryptotanshinone (*CT*), dihydrotanshinone I (*DiHTs I*) and tanshinone II A (*TsII A*), as well as the total contents of hydrophilic compounds and lipophilic compounds were calculated. The UPLC system (Waters, Milford, MA) was equipped with a quaternary solvent manager, an auto sampler manager and a photodiode array (PDA) detector connected to Waters Empower 2 software. The chromatographic separation was performed on a Waters Acquity UPLC BEH C 18 column (2.1 ×100 mm, 1.7 μm). The extraction method and chromatographic conditions followed Liu’s method [28]. The HPLC-grade acetonitrile was purchased from Fisher Scientific (NJ, USA). The water was *Wahaha* purified water purchased from Hangzhou *Wahaha* Group. Phosphoric acid and methanol of analytical grade were obtained from Beijing Beihua Fine Chemicals Co. Ltd. (Beijing, China). The standard compounds of *Sal B*, *Ts I*, *CT* and *TsII A* were purchased from the National Institutes for Food and Drug Control, Beijing, China, and *Dss*, *Ra* and *DiHTs I* were purchased from Chengdu Must Bio-Technology, Chengdu, China. Statistical analysis was carried out by the SPSS 19.0. The statistical significance of differences was evaluated by Student’s *t*-test to compare the mean value. *P* value of <0.05 was considered to be statistically significant.

### Comparison of consistency with production germplasms

The fingerprint was analyzed by UPLC using the above method. The similarities of entire chromatographic patterns among the tested samples were calculated by SA using the professional software of Similarity Evaluation System for Chromatographic Fingerprint of Traditional Chinese Medicine (Version 2004 A) recommended by the Chinese Pharmacopoeia Committee. After analyzing a group of chromatograms, a simulated mean chromatogram was generated as a representative standard chromatographic profile by SA. Then the correlation coefficient of similarity between each pair of chromatograms was calculated.

## Results

### Performance of ZD1 and ZD2 in Beijing

#### Plant characteristics

**Fig 1:**
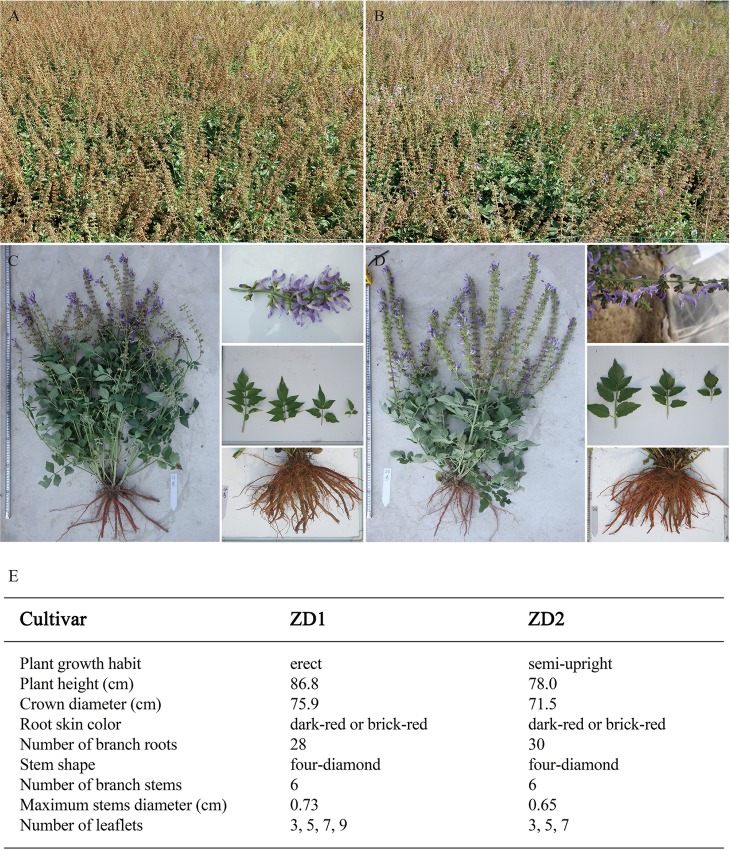
The plant characteristics of ZD1 and ZD2 in mid-June (C and D) and mid-Sep 2013 (A and B). The data of plant characteristics was shown in Fig 1E. The seeds were sown in a field at IMPLAD, Beijing and subsequently transplanted in April 2013.

The plants of ZD1 stood erect and showed great vigor, with an average height of about 86.8 cm and a crown diameter of about 75.9 cm (Fig 1A). The root skin color was dark-red or brick-red, and each root had 28 branch roots on average. The stem was four-diamond shaped, and each plant had about six branch stems with the maximum diameter of 0.73 cm. The leaf was pinnately a compound with 3–9 leaflets (Fig 1C). The plants of ZD2 were semi-upright and showed great growth vigor, with an average height of about 78.0 cm and a crown diameter of about 71.5 cm (Fig 1B). The root skin color was the same as that of ZD1, and each root had about 30 branch roots. The maximum diameter of ZD2 stems was 0.65 cm. Different from ZD1, ZD2 had leaves with 3–7 leaflets (Fig 1D).

#### Yield evaluation

**Fig 2:**
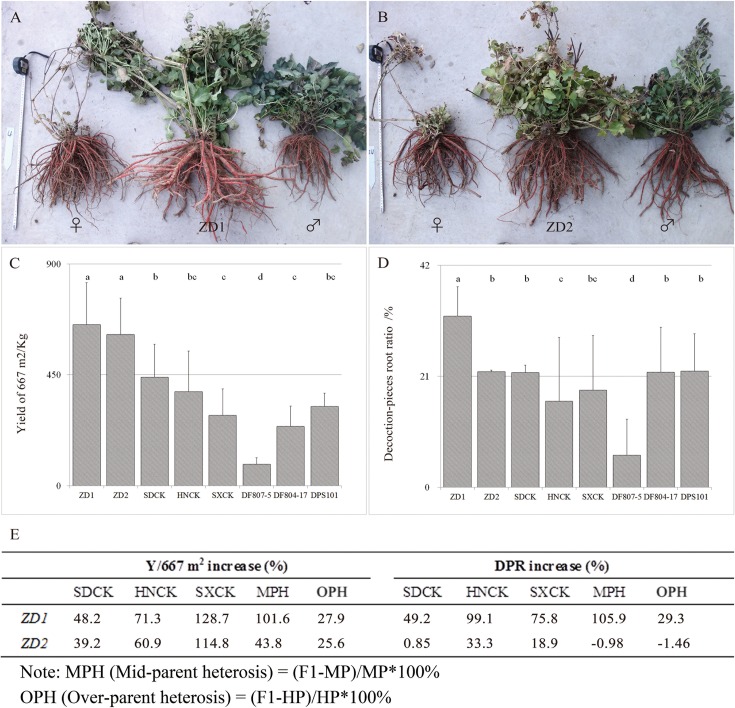
The yield characters of ZD1 and ZD2. The seeds were sown in a field at IMPLAD, Beijing, and subsequently transplanted in April 2013. A, B: the roots morphology of ZD1, ZD2 and their parents, respectively; C, D: the yield of 667 m^2^ (Y/667 m^2^) and decoction-piece root ratio (DPR) of ZD1 and ZD2 compared with the controls and their parents. Values followed by different letters are significantly different (P <0.05) among cultivars according to the Tukey-Kramer test. E: The over-standard heterosis, mid-parent heterosis (MPH) and over-parent heterosis (OPH) analysis of ZD1 and ZD2.

Because *Danshen* is usually used for both component extraction and decoction pieces, Y/667 m^2^ and DPR were simultaneously measured to evaluate the yield characteristics of the cultivars. In the two-year comparison, both ZD1 and ZD2 showed obvious advantages in the root weight. ZD1 had a yield of 653.7 kg/667 m^2^, being 48.2% higher than that of SDCK, 71.3% higher than that of HNCK, and 128.7% higher than that of SXCK. While ZD2 had a yield of 614.0 kg/667 m^2^, being 39.2% higher than that of SDCK, 60.9% higher than that of HNCK, and 114.8% higher than that of SXCK (Fig 2A–2D). ZD1 had a DPR of 32.4%, being 49.2% higher than that of SDCK, 99.1% higher than that of HNCK, and 75.8% higher than that of SXCK. While ZD2 had a DPR of 21.9%, which was not significantly higher than that of SDCK but significantly higher than those of HNCK and SXCK. ZD1 plants showed both increased yield and DPR compared with their parents, resulting in MPH (middle-parent-heterosis) values up to 101.6% and 105.9%, respectively; while ZD2 plants displayed an increased yield by 43.8% compared with the mean value of both parents. The OPH (over-parent heterosis) of ZD1 and ZD2 was respectively 27.9% and 25.6% in terms of yield (Fig 2E).

#### Measurement of active compounds content

**Fig 3:**
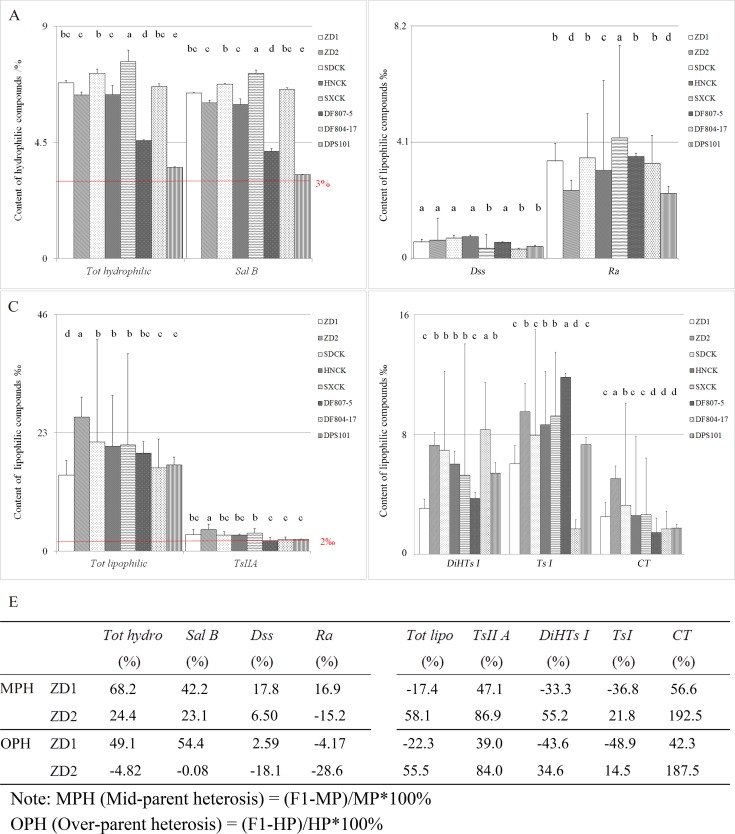
The active compounds content of ZD1 and ZD2. Seeds were sown in a field at IMPLAD, Beijing, and subsequently transplanted in April 2013. A, B: The hydrophilic compounds (including *Sal B*, *Dss*, and *Ra*) of ZD1, ZD2 and their parents, respectively; C, D: The lipophilic compounds (including *TsII A*, *Ts I*, *CT*, and *DiHTs I*.) of ZD1 and ZD2 compared with the controls and their parents. Values followed by different letters are significantly different (P <0.05) among cultivars according to the Tukey-Kramer test. E: The MPH and OPH analysis of ZD1 and ZD2. *Dss*: danshensu, *Ra*: rosmarinic acid, *Sal B*: salvianolic acid B, *Ts I*: tanshinone I, *CT*: cryptotanshinone, *DiHTs I*: dihydrotanshinone I, *TsII A*: tanshinone II A, *Tot hydro*: total content of hydrophilic compounds, *Tot lipo*: total content of lipophilic compounds.

The contents of the important medical compounds of *Danshen* were measured, including the hydrophilic compounds, *Dss*, *Ra* and *Sal B*, and the lipophilic compounds, *TsII A*, *Ts I*, *CT*, and *DiHTs I*. In some *Danshen*-based preparations, water, alcohol and alcohol-water were used as solvent for different active compound extraction according to *The Chinese Pharmacopoeia* and a certain type of ingredient was extracted. When used as decoction pieces, mainly hydrophilic compounds were extracted. In *The Chinese Pharmacopoeia* only *Sal B* and *TsII A* of *Danshen* are given the standard contents of no less than 3.0% and 2.0‰, respectively. For ZD1, the average contents of salvianolic acid B and tanshinone II A were respectively 6.4% and 3.2‰, 2.14 and 1.59 times of the required values by *The Chinese Pharmacopoeia*. For ZD2, the average content of salvianolic acid B was 6.0%, 2.01 times of the required value by *The Chinese Pharmacopoeia*, and that of tanshinone II A was 4.2‰, 2.09 times of the required value by *The Chinese Pharmacopoeia* and 29.6% higher than the average content of 3CKs. In addition, the highest contents of all lipophilic components were detected in ZD2 (Fig 2C and 2D). For *Tot lipo*, *DiHTs I*, *Ts I and CT*, the content of ZD2 were 28.2%, 19.4%, 10.6% and 78.7% higher than the average content of 3CKs. It’s worth noting that the content of lipophilic compounds in ZD2 was significantly higher than ZD1, which shared the same female parent. Heterosis analysis showed that the cross combination of ZD1 possessed a positive heterosis (mid-parent heterosis and over-parent heterosis) in *Tot hydro*, *Dss*, *Sal B*, *TsII A*, *CT*. While the cross combination of ZD2 showed a pervasive heterosis (mid-parent heterosis and over-parent heterosis) in all tested lipophilic compounds content (Fig 3E). The trend of active compounds concentrate change of two varieties, indicated that strong heterosis varieties with high hydrophilic or lipophilic compounds content can obtain through reasonable hybrid combination.

#### Comparison of the consistency with production germplasms

**Fig 4:**
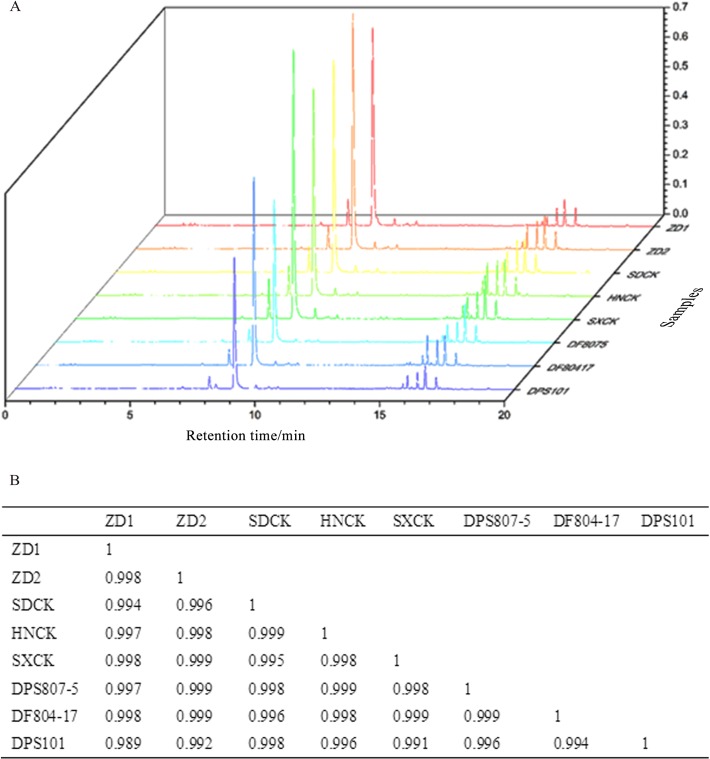
Comparison of the consistency of production germplasms. A: The UPLC fingerprint of ZD1 and ZD2 compared with the controls and their parents; B: The SA of ZD1 and ZD2 compared with the controls and their parents.

To elucidate whether hybridization would change the components of these plants, the chemical fingerprint was assayed among the hybrids, their parents and commonly cultivated controls (Fig 4A). As shown in Fig 4B, the correlation coefficients for the similarity of UPLC fingerprint were >0.989, indicating that the compositions of different hybrid cultivars were almost the same as those of the controls, although the contents of some compounds varied to some extent.

#### Performance of ZD1 and ZD2 in *Danshen*-producing areas

**Fig 5:**
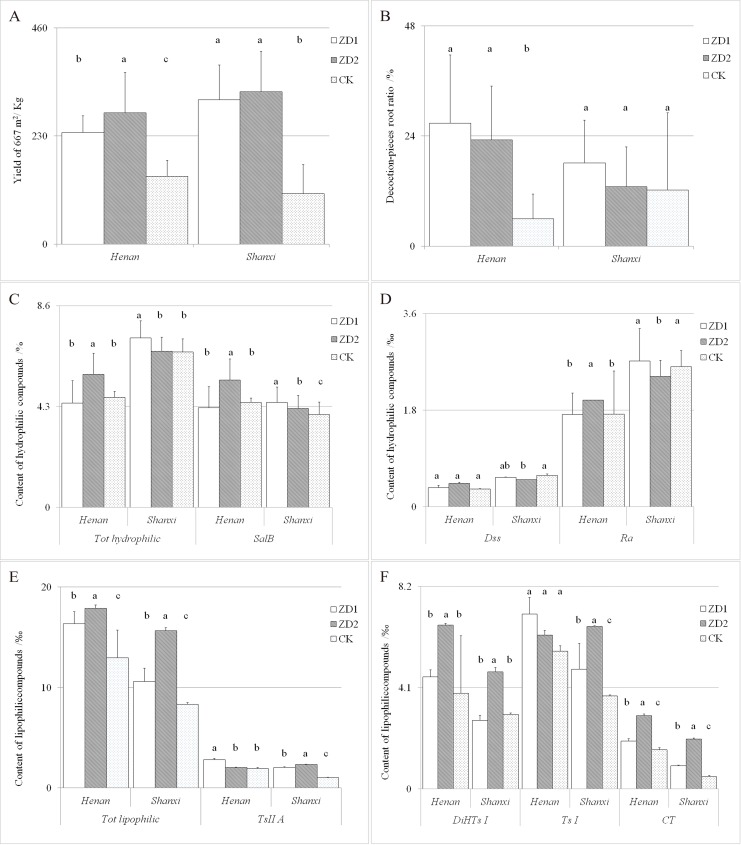
The Performance of ZD1 and ZD2 planted in the provinces of Henan and Shanxi. Values followed by different letters are significantly different (P <0.05) among cultivars according to the Tukey-Kramer test. A, B: The yield of 667 m^2^ (Y/667 m^2^) and decoction-piece root ratio (DPR) of ZD1 and ZD2 and local production control; C, D: The hydrophilic compounds of ZD1 and ZD2 and local production control; E, F: The lipophilic compounds of ZD1 and ZD2 and local production control. *Dss*: danshensu, *Ra*: rosmarinic acid, *Sal B*: salvianolic acid B, *Ts I*: tanshinone I, *CT*: cryptotanshinone, *DiHTs I*: dihydrotanshinone I, *TsII A*: tanshinone II A, *Tot hydro*: total content of hydrophilic compounds, *Tot lipo*: total content of lipophilic compounds.

ZD1 and ZD2 were further planted in Henan and Shanxi, the major *Danshen*-producing areas in China. Both hybrid cultivars showed high yield potential in the two provinces, but a better performance in Shanxi if only the total root yield was considered. The Y/667 m^2^ of ZD1 and ZD2 was respectively 306.9 kg and 324.2 kg, increasing by 186.4% and 202.5% compared with the controls (Fig 5A, Table 1). However, when compared with the controls in terms of the DPR, ZD1 showed a slight advantage while ZD2 showed no advantage in Shanxi; in Henan, ZD1 and ZD2 increased by 384.4% and 287.9%, respectively (Fig 5B, Table 1). For medicinal purposes, the contents of the bioactive components should receive attention. The contents of *Sal B* and *TsII A* in the ZD1 and ZD2 cultivars planted in Henan and Shanxi reached the standards of the Chinese Pharmacopoeia compared with the controls, which show *TsII A* contents below the standard in both planting areas, particularly in Shanxi (only 1.04‰). The *Sal B* content of ZD2 in Henan was obviously higher than that of the control and ZD1 (5.43%, 21.07% higher than the control). Furthermore, the content of lipophilic compounds in ZD2 was relatively stable and higher in Henan and particularly in Shanxi (Fig 5C and 5D, Table 1). Nevertheless, multi-area testing further demonstrated that ZD1 has an advantage in yield, DPR and hydrophilic compounds content, while ZD2 has an advantage in yield and lipophilic compounds content.

**Table 1:** The over-standard heterosis of ZD1 and ZD2 compared with the local production controls planted in Henan and Shanxi provinces.

Location	Cultivar	Y/667m^2^(%)	DPR(%)	*Tot hydro*(%)	*Sal B*(%)	*Dss*(%)	*Ra*(%)	*Tot lipo*(%)	*TsIIA*(%)	*DiHTs I*(%)	*Ts I*(%)	*CT* (%)
Henan	*ZD1*	64.7	348.4	20.9	21.1	9.09	-0.58	26.1	44.6	17.1	26.9	22.2
	*ZD2*	93.9	287.9	-5.24	-5.53	33.3	15.0	37.8	5.64	71.3	11.7	87.3
Shanxi	*ZD1*	186.4	48.2	12.1	12.9	-6.78	3.85	27.2	96.2	-8.28	28.7	86.0
	*ZD2*	202.5	6.04	5.05	6.16	-13.6	-7.7	88.2	124.0	57.0	75.0	302.0

## Discussion

The male sterile line, as the most effective pollination control system, has been widely used in cross-breeding of crops and vegetables. In this study, several steady sterility male-sterile lines were developed through successive testcrossing, and two hybrid cultivars were obtained, excelling in the yield and active compounds content compared with the cultivated types in the *Danshen*-producing areas of China. The UPLC fingerprint indicated that hybridization only changed the concentration of chemical compounds rather than the composition. This is the first report on the heterosis breeding for *S*. *miltiorrhiza*, which may lay a foundation of medicinal plant breeding that uses vegetative organs, especially roots as economic organs. The wide cultivation of cultivars ZD1 and ZD2 will largely promote the *Danshen* production, making it possible to establish standards and control on *Danshen* quality based on the consistent, stable and controllable characterisics of hybrid cultivars.

*Danshen* is usually used for both component extraction and decoction pieces, when used as component extraction, culture with high yielding and total hydrophilic or lipophilic components were selected. While for decoction pieces type culture, character of yield, DPR and total hydrophilic should be considered. The yield and bioactive component content usually vary greatly in different planting environments and under different managements. In the present study, ZD1 and ZD2 had varied yields when planted in Beijing, Henan and Shanxi. Generally speaking, however, their yields were significantly higher than that of the local cultivated types. More importantly, the contents of the core bioactive components of both ZD1 and ZD2 exceeded the standard set by *The Chinese Pharmacopoeia* in Beijing, Henan and Shanxi. ZD2 showed obvious advantages in terms of bioactive component content, although some tested compounds of ZD2 showed lower contents than the controls, such as the contents of *Dss* (0.51‰, lower by 13.6%) and *Ra* (2.43‰, lower by 7.69%) in Shanxi. Collectively, ZD1 is a broadly adaptive cultivar with advantages in yield, DPR and total hydrophilic compound content, and is suitable for different medicinal purposes (decoction-pieces and extraction); while ZD2 is a high-yield cultivar with obvious advantages in lipophilic compounds and is applicable to lipophilic component extraction. In addition, the upright plant growth of ZD1 would favor the daily management of farmers, and a small plant model of ZD2 may enable increased production through rational close planting. Further exploration on the most suitable planting management for ZD1 and ZD2 in different producing areas will help realize the yield and quality potential of hybrid cultivars ZD1 and ZD2.

In this study, ZD1 showed the highest DPR, indicating that ZD1 had the thickest branch roots among the test types. ZD1 had relatively high contents of hydrophilic compounds, especially the main component *Sal B*; while ZD2 had relatively high contents of lipophilic compounds, in agreement with previous reports that the bioactive lipophilic compounds are mainly distributed in the bark, and the hydrophilic compounds are distributed in all tissues of the root [29, 30]. The branch roots of ZD2 with lower DPR were thinner but larger in number, thus resulting in a higher percent of the bark in the root, which ultimately led to the relatively high contents of lipophilic compounds.

It has been reported that the interspecies hybridization would change the composition and proportion of F1 [31, 32], but no report concerns the variation of chemical properties in intraspecific hybrid cultivars. In this study, although the internal quality was compared between production germplasms and hybrid cultivars using UPLC fingerprint, the pharmacodynamics was not evaluated for the safety reason. Moreover, the low seed production speed limited the area expansion of test plots, which might further affect the promotion of these two cultures. Therefore, the development of a more effective method of pollination would be of great significance in the future research.

## Conclusion

In this study, two hybrid cultivars “Zhongdanyaozhi No. 1” and “Zhongdanyaozhi No. 2” were compared with three widely accepted types in yield, DPR and active compounds content. The yield of ZD1 and ZD2 exceeded the three CKs by more than 39.2%. The composition of the two hybrid cultivars was similar to the control, although the content of some compounds varied to some extent. ZD1 is a high yielding and thick-root-type cultivar which is suitable for decoction pieces; while ZD2 is suitable for component especially lipophilic component extraction. Hybridization is a useful method to promote the quality of *S*. *Miltiorrhiza* germplasm.
